# Assessing HIV/AIDS Awareness and the Factors Influencing It in a High HIV Prevalence District: Insights From a Large-Scale Community Survey Among the Rural Population

**DOI:** 10.7759/cureus.85648

**Published:** 2025-06-09

**Authors:** Mahesh Singiri, Anil R, Vignesh Jambulingam, Praveen Kumar BA, Chitra Nagaraj, Janakiraman Pichandi

**Affiliations:** 1 Community Medicine, People’s Education Society Institute of Medical Sciences and Research, Kuppam, IND; 2 Preventive Medicine, People’s Education Society Institute of Medical Sciences and Research, Kuppam, IND; 3 Preventive Medicine, People's Education Society Institute of Medical Sciences and Research, Kuppam, IND; 4 Biostatistics, People’s Education Society Institute of Medical Sciences and Research, Kuppam, IND

**Keywords:** community-based study, hiv/aids, hiv-kq 18, knowledge assessment, rural-based population

## Abstract

Introduction

HIV/AIDS is one of the complex diseases of today’s world, with 39 million people living with HIV (PLHIV). India has the second largest number (2.4 million) of PLHIV/AIDS. Chittoor is a district with a high prevalence (0.5%) of HIV in the state of Andhra Pradesh. An important component of the HIV control program is the awareness among people about HIV.

Objectives

The objectives of this study are to assess knowledge of HIV/AIDS among the general population in the rural community and to identify the factors associated with knowledge of HIV among them.

Materials and methods

This is a large community-based cross-sectional study done in the rural community of Chittoor district, India, between December 2022 and July 2023. Using a cluster sampling technique, 4458 participants were included in the study. After obtaining informed consent, a face-to-face personal interview was conducted at their household using a validated HIV Knowledge Questionnaire (HIV-KQ-18). Data were analyzed using IBM SPSS Statistics, version 23.0 (IBM Corp., Armonk, NY).

Results

Among the 4458 participants, 66.8% exhibited poor knowledge, 28.7% had moderate knowledge, and 4.5% possessed good knowledge of HIV/AIDS. Notably, factors such as age, gender, education, marital status, occupation, and socioeconomic status showed significant associations with HIV/AIDS knowledge (p-value < 0.05). It was concerning that only one-fifth of the participants had attended at least one awareness session on HIV/AIDS, and an alarming three-fourths had never engaged in discussions about HIV/AIDS with anyone. This highlights a substantial gap in awareness and communication surrounding HIV/AIDS within the rural community.

Conclusion

The level of awareness regarding HIV/AIDS in rural communities is insufficient. To address this gap, there is a pressing need for innovative and comprehensive dissemination of scientific information specifically tailored for the rural population. By providing better education and knowledge, we can effectively reduce the transmission of HIV within these communities and contribute to global efforts in combating this pandemic.

## Introduction

The HIV/AIDS epidemic has devastated many individuals, families, and communities, affecting both industrialized and developing countries [1]. AIDS is not only a complex disease but also a social, economic, cultural, and political problem.

HIV/AIDS is a complex disease and one of the common health problems of today’s world [2]. According to the Joint United Nations Program on HIV/AIDS (UNAIDS) Fact Sheet 2024, 39.9 million people living with HIV (PLHIV) and 42.3 million people have died of AIDS-related illnesses since the start of the epidemic [3,4].

In India, the number of PLHIV is estimated to be 2.54 million, with an adult HIV prevalence of 0.20% [5] (males: 0.22% and females: 0.20%). India ranks third globally in terms of the total number of PLHIV. Among Indian states, Andhra Pradesh (AP) has the second highest number of HIV cases, with an adult prevalence of 0.67% [6]. High prevalence among the age group of 15-49 years indicates that AIDS continues to impact individuals in the prime of their working lives [7].

AP is one of the high prevalence states in India. Within AP, Chittoor district was identified as one of the high-burden districts, with an adult prevalence exceeding 1%. Chittoor district is home to 18,234 PLHIV/AIDS [8]. Adolescents are susceptible to unhealthy practices due to a lack of knowledge and curiosity to try new things [9].

There are several reasons for the high prevalence of HIV infection among the general population, particularly youth in rural areas, and one of them could be inadequate and inaccurate information about the modes of transmission of HIV due to cultural or religious beliefs or lack of education [10].

Despite advancements in treatment and prevention, HIV-related stigma and discrimination persist as significant barriers in the effective fight against the HIV/AIDS epidemic.

Lack of awareness would make people hesitant to get the test done; therefore, more PLHIV are unaware that they are suffering from HIV/AIDS, which increases the chance that his or her contacts would contract the infection as a result of not taking the necessary precautions [11]. Addressing these social and structural challenges is crucial for reducing transmission rates and improving the overall well-being of individuals living with HIV/AIDS.

The prevalence of HIV in India is steadily declining from 0.54% in the early 2000s to 0.22% in 2020. Though this is a positive result, some states like AP have been estimated to have adult HIV prevalence higher than the national average, which is a matter of concern. However, according to the NFHS-4 and NFHS-5 data, there is a mild increase in knowledge among the Indians, which can be correlated to the positive results achieved. The major challenges or controversies found in rural India that hinder the HIV awareness activities are as follows: (a) lack of support given to comprehensive sex education, (b) high level of stigma and discrimination among the affected individuals and their family members, (c) limited health care support pertaining to HIV/AIDS in particular, and (d) mental health issues associated with HIV/AIDS.

Knowledge is a key component of HIV risk reduction programs. Even though the state of AP has the second highest number of reported HIV cases, HIV/AIDS continues to be a serious public health issue in rural areas of India, with high prevalence, where awareness and health literacy are often low. Although national surveys show differences across regions, there is little community-level information about HIV knowledge and related socio-demographic factors. This study was carried out to address this gap and help design targeted HIV prevention strategies suited to local needs.

## Materials and methods

A community-based cross-sectional study was conducted among 4458 study subjects of Kuppam Mandal, Chittoor district, India, from December 2022 to July 2023.

A cluster sampling method was employed for selecting study participants. In the Kuppam Mandal, there are a total of 63 revenue villages, each of which was considered as a cluster for the purpose of the study. To ensure an adequate population base within each cluster, villages with a population exceeding 1500 individuals were first identified; a total of 30 villages met this criterion. From these 30 eligible villages, 10 villages were selected using a simple random sampling (SRS) technique.

The sample size was calculated by assuming the prevalence of poor knowledge on HIV/AIDS among the participants as 50% and an error of 2.5%. The formula n = 4pq/l^2^ was used, and the sample size obtained was 3520, considering a design effect of 2 (cluster sampling technique) and an expected non-response rate of 10%. From the 10 villages selected, approximately 352 participants were proportionately sampled from each village to achieve the total intended sample size. During the house visit, if more than one adult was present in any house, all of them were included in the study; thus, a sample of 4458 subjects was included in this study. Subjects above 18 years were included in the study. Those who have been diagnosed with chronic diseases and other mental illnesses were excluded.

The study participants received a thorough explanation of the study purpose, and their informed consent was obtained. A face-to-face interview was conducted, and information was gathered using a semi-structured questionnaire in private. The questionnaire had two sections: (a) socio-demographic characteristics and (b) HIV-KQ-18 questionnaire [12]. The “HIV Knowledge Questionnaire” is a validated questionnaire designed to assess the knowledge necessary for HIV prevention. Respondents are presented with 18 statements about HIV and are asked to indicate whether they believe each statement is true or false, or if they are unsure (“don’t know”). Correct answers were scored “1,” while incorrect responses and “don’t know” responses were scored as “0” (Table 1).

**Table 1:** Scoring for the HIV Knowledge Questionnaire ranging from 0 to 18

Score	Knowledge level
0-6	Poor
7-12	Moderate
13-18	Good

The data obtained were analyzed using IBM SPSS Statistics, version 23.0 (IBM Corp., Armonk, NY). Categorical data were analyzed using percentages and proportions. Continuous data were analyzed using mean and standard deviation. For inferential statistics, data were analyzed using the chi-square test. A p-value of <0.05 was considered statistically significant.

## Results

Among the total of 4458 study participants, the majority belonged to the age group of 31-40 years (1205, 27.03%). The mean age of study participants was 38.6 ± 13.6 years. It was noted that 53.3%, 46.6%, and 0.1% were males (2378), females (2077), and transgenders (three), respectively. Most (2945, 66.07%) of the subjects were employed, and the majority belonged to the middle (1181, 26.5%) and lower middle class (1351, 30.3%). The majority (2884, 64.7%) of study participants were literate, and most (3695, 82.9%) of them were married, as shown in Table 2.

**Table 2:** Socio-demographic profile of study participants ^#^The data have been represented as n (number) and % (percentage). ^*^Modified BG Prasad classification for 2022

Characteristics	Frequency, n (%)^#^
Age	
<20 years	333 (7.4)
21-30 years	1165 (26.1)
31-40 years	1205 (27.03)
41-50 years	910 (20.4)
>51 years	845 (18.9)
Gender	
Male	2378 (53.3)
Female	2077 (46.5)
Transgender	3 (0.07)
Occupation	
Employed	2945 (66.1)
Unemployed	1513 (33.9)
Socioeconomic status*	
Upper class	447 (10.0)
Upper middle class	843 (18.9)
Middle class	1181 (26.5)
Lower middle	1351 (30.3)
Lower class	636 (14.3)
Education	
Illiterate	1574 (35.3)
Literate	2884 (64.7)
Marital status	
Unmarried	713 (15.9)
Married	3695 (82.9)
Divorced	50 (0.01)
Total	4458 (100%)

**Figure 1:**
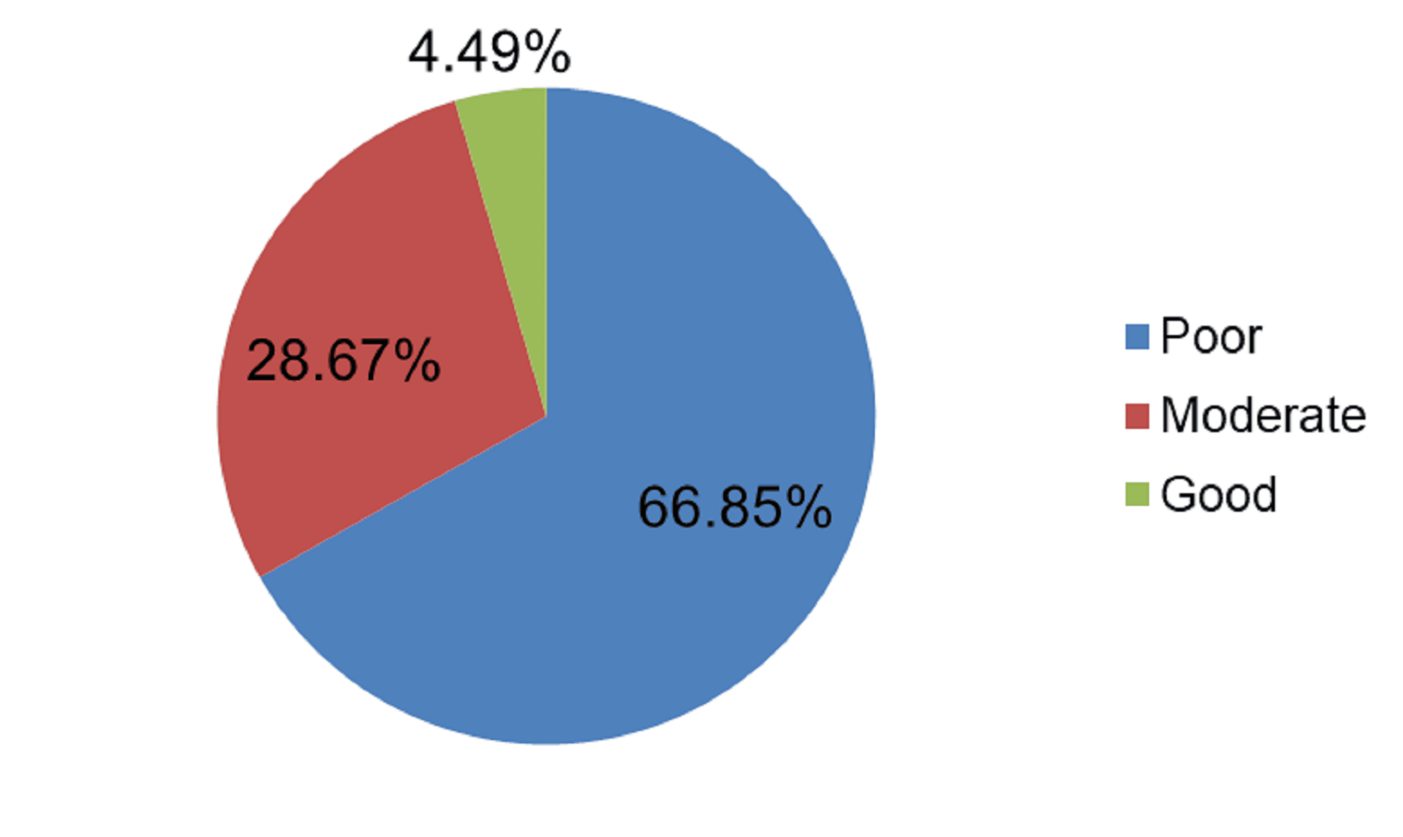
Knowledge of HIV/AIDS among the study participants The data have been represented as % (percentage).

Out of the total 4458 participants, 66.8% (2980) demonstrated poor knowledge regarding HIV/AIDS, as shown in Figure 1.

It was found that among all the age groups, more than 50% of the study participants had poor knowledge, and the poor level of knowledge was higher among the older age groups. About 31.3% (922) of unemployed study subjects had moderate and good knowledge when compared to 36.8% (556) of the employed; more than 75% of the study population of lower socioeconomic class (IV & V) had poor knowledge. About 40% (1172) of the literate showed moderate and good knowledge, which dropped to half (306, 20%) among the illiterates.

About 53.7% (383) of unmarried participants had better knowledge than the married (1082, 29.3%) and the divorced group (13, 26%).

The socio-demographic factors, such as age, gender, occupation, education, socioeconomic status, and marital status, were found to be statistically significantly associated with knowledge of HIV/AIDS (p-value < 0.05), as shown in Table 3. A p-value of <0.05 was considered statistically significant.

**Table 3:** Association between socio-demographic determinants and knowledge about HIV/AIDS The data have been represented as n (number) and % (percentage). ^#^A p-value of <0.05 was considered statistically significant. ^*^Statistically significant.

Characteristics	Knowledge, n (%)			Chi-square score (p-value)^#^
	Poor, n (%)	Moderate, n (%)	Good, n (%)	
Age (years)				
<20	168 (50.5%)	153 (45.9%)	12 (3.6%)	212.15 (0.000)*
21-30	656 (56.3%)	427 (36.6%)	82 (7.0%)	
31-40	804 (66.7%)	338 (28.0%)	63 (5.2%)	
41-50	666 (73.1%)	210 (23.0%)	34 (3.7%)	
>51	686 (81.1%)	150 (17.7%)	9 (1.0%)	
Gender				
Male	1557 (65.5%)	688 (28.9%)	133 (5.6%)	17.62 (0.001)*
Female	1422 (68.5%)	588 (28.3%)	67 (3.2%)	
Occupation				
Unemployed	2023 (68.7%)	779 (26.5%)	143 (4.8%)	22.92 (0.001)*
Employed	957 (63.2%)	499 (32.9%)	57 (3.9%)	
Socioeconomic status				
I, II, and III	1983 (62.5%)	1007 (31.8%)	178 (5.7%)	98.04 (0.001)*
IV and V	997 (77.3%)	271 (21.0%)	22 (1.7%)	
Education				
Illiterate	1268 (80.5%)	284 (18.0%)	22 (1.5%)	215.9 (0.001)*
Literate	1712 (59.3%)	994 (34.5%)	178 (6.2%)	
Marital status				
Unmarried	330 (46.3%)	328 (46.0%)	55 (7.7%)	162.87 (0.001)*
Married	2613 (70.7%)	938 (25.4%)	144 (3.9%)	
Divorced	37 (74.0%)	12 (24.0%)	1 (2.0%)	

The majority (3499, 78.48%) of study subjects never attended any awareness class on HIV/AIDS, and most (3328, 74.67%) of them never discussed HIV/AIDS with anyone, as shown in Table 4.

**Table 4:** Distribution of study participants based on exposure to HIV/AIDS awareness activities and discussions The data have been represented as n (number) and % (percentage).

Question	Yes, n (%)	No, n (%)	Total, n (%)
Have you ever attended any awareness classes on HIV/AIDS?	959 (21.52)	3499 (78.48)	4458 (100)
Have you ever discussed HIV/AIDS with anyone?	1130 (25.34)	3328 (74.67)	4458 (100)

## Discussion

Understanding the current status of HIV/AIDS knowledge among rural populations is essential for designing effective public health interventions. Despite decades of national awareness campaigns under programs like the National AIDS Control Program (NACP), gaps in understanding continue to persist in rural areas, especially among low-income groups. This community-based cross-sectional study, carried out among 4,458 participants in Kuppam Mandal, Chittoor district, India, offers important insights into these ongoing challenges and highlights areas for targeted health education.

Among the study participants, the majority were aged between 31 and 40 years (27.03%), male (53.34%), married (82.88%), employed (66.07%), and from lower middle or lower socioeconomic backgrounds (44.6%). These findings are in line with previous studies from rural India. Research by Yadav et al. [13] in Gujarat observed a largely male and lower socioeconomic youth population, while Singh and Jain [14] found similar socio-demographic patterns among rural adolescents. Easwaran et al. [1] in South India also described comparable rural populations, reinforcing the observation that social factors such as education and income play a major role in shaping health awareness.

The main findings revealed that 66.8% of the respondents had poor knowledge of HIV/AIDS, and only 4.5% demonstrated good knowledge, despite ongoing government-led efforts. This underlines a persistent gap in awareness at the community level. In comparison to global benchmarks, such as Carey and Schroder’s HIV-KQ-18 study in the United States [12], where higher understanding was recorded, rural India still shows significant shortfalls in both basic and detailed knowledge about HIV/AIDS. In contrast, studies conducted in rural India have highlighted significant gaps in HIV-related knowledge. For instance, a study assessing awareness among rural youth in the Saurashtra region of Gujarat found that only 59% had heard of HIV/AIDS, and less than 50% could correctly identify modes of transmission [13]. Another study focusing on rural adolescents in Maharashtra reported that while 71.5% recognized that a faithful uninfected partner reduces HIV risk, misconceptions persisted, with 46.7% believing mosquito bites could transmit HIV [15]. This poor level of knowledge observed in this study could be due to the stigma associated with the disease, especially in the study area. As well as there is no proper sex education curriculum integrated into the school curriculum, which makes it harder for adolescents and youth to get validated information surrounding the disease. And the majority of the study population were from lower economic status, which questions their ability for quality education and their access to information.

Several factors were found to significantly influence the level of HIV/AIDS knowledge. Older participants had lower awareness, with more than 80% of individuals over 50 years showing poor knowledge, similar to trends reported by Malleshappa et al. [7]. Educational status had a strong impact; only 20% of illiterate individuals had moderate or good knowledge compared to 40% among literates, reflecting findings from Ganesan et al., in Nellore, India [2]. Employment and socioeconomic status also played important roles, with employed individuals and those from higher income groups showing better knowledge levels. Interestingly, unmarried participants had higher levels of awareness compared to married participants, likely due to more recent exposure to school-based or peer-driven information sources.

Another important observation was the low proportion of individuals who had ever attended an HIV/AIDS awareness session (20%) or discussed the topic with others (25%). These figures suggest that cultural stigma, community taboos, and a lack of consistent health communication strategies are key barriers to improving knowledge. Even among those who were literate or employed, these social obstacles appear to limit information sharing and understanding. This points to the urgent need for localized, continuous, and culturally appropriate health education efforts in rural areas. Building stronger community networks, integrating HIV/AIDS discussions into routine public health outreach, and using peer-led interventions could help bridge these knowledge gaps and improve overall community awareness. 

Limitations of the study

Responses could have been influenced by social desirability bias, especially given the sensitive nature of HIV/AIDS. This study assessed knowledge but did not comprehensively evaluate attitudes or actual practices, which are equally important in HIV prevention. Although a cluster sampling technique was used, certain high-risk populations (e.g., migrants, MSM, and PLHIV) may have been underrepresented. This study was done among the rural population, which could restrict its generalizability to urban and peri-urban areas. The study did not collect data on the type, duration, or quality of HIV/AIDS awareness sessions attended, which limits interpretation of their impact on knowledge.

## Conclusions

This study revealed concerning trends regarding HIV/AIDS knowledge and awareness. A majority of participants had poor knowledge of HIV/AIDS, highlighting a significant awareness gap. The risk factors associated with poor knowledge of HIV/AIDS were older age group, male gender, illiteracy, unemployment, lower socioeconomic status, and married status. This highlights the importance of considering these risk factors in planning HIV/AIDS education and awareness campaigns.

The findings highlight the need for targeted HIV/AIDS education to bridge knowledge gaps and improve community communication. Regular health education sessions, role plays, and focus group discussions can raise awareness, especially among rural youth. Emphasizing HIV issues helps protect individuals and reduce stigma. Establishing psychosocial support networks and support groups is vital for the holistic care of PLHIV. Public enlightenment programs are crucial to spread accurate information and dispel myths in rural areas.

## Appendices

**Table 5:** HIV-KQ-18: questionnaire items Source article: reference [12].

HIV-KQ-18: questionnaire items
Response: true (T), false (F), and don't know (DK)
1. Coughing and sneezing do not spread HIV. (T/F/DK)
2. A person can get HIV by sharing a glass of water with someone who has HIV. (T/F/DK)
3. Pulling out the penis before a man climaxes/cums keeps a woman from getting HIV during sex. (T/F/DK)
4. A woman can get HIV if she has anal sex with a man. (T/F/DK)
5. Showering or washing one's genitals/private parts after sex keeps a person from getting HIV. (T/F/DK)
6. All pregnant women infected with HIV quickly show serious signs of being infected. (T/F/DK)
7. People who have been infected with HIV quickly show serious signs of being infected. (T/F/DK)
8. There is a vaccine that can stop adults from getting HIV. (T/F/DK)
9. People are likely to get HIV by deep kissing, putting their tongue in their partner’s mouth, if their partner has HIV. (T/F/DK)
10. A woman cannot get HIV if she has sex during her period. (T/F/DK)
11. There is a female condom that can help decrease a woman's chance of getting HIV. (T/F/DK)
12. Using a condom during sex prevents a person from getting HIV. (T/F/DK)
13. A person will not get HIV if she or he is taking antibiotics. (T/F/DK)
14. Having sex with more than one partner can increase a person's chance of being infected with HIV. (T/F/DK)
15. Taking a test for HIV one week after having sex will tell a person if she or he has HIV. (T/F/DK)
16. A person can get HIV by sitting in a hot tub or a swimming pool with a person who has HIV. (T/F/DK)
17. A person can get HIV from oral sex. (T/F/DK)
18. Using Vaseline or baby oil with condoms lowers the chance of getting HIV. (T/F/DK)

## References

[1] Easwaran V, Nayakanti D, Mohammed JS, Gerardo AU (2011). Assessment of knowledge about HIV/AIDS among public: a rural perspective of South India. Asian J Pharm Health Sci.

[2] Ganesan V, Chandrasekhar V, Raghavendra P, Rushender R (2017). A study on awareness of HIV/AIDS and attitude toward people living with HIV/AIDS among engineering college students of Nellore district, Andhra Pradesh, India. Int J Community Med Public Health.

[3] (2024). UNAIDS Fact Sheet 2024. https://www.unaids.org/sites/default/files/media_asset/UNAIDS_FactSheet_en.pdf.

[4] (2024). World Health Organization. HIV data and statistics. https://www.who.int/teams/global-hiv-hepatitis-and-stis-programmes/hiv/strategic-information/hiv-data-and-statistics.

[5] (2024). National AIDS Control Organization. India HIV estimates 2023: technical report. https://naco.gov.in/sites/default/files/India%20HIV%20Estimates%202023_Technical%20Report_Final_17%20DEC%202024%20%281%29.pdf.

[6] (2022). National AIDS Control Organization. India HIV estimates 2021: fact sheet. https://naco.gov.in/sites/default/files/India%20HIV%20Estimates%202021%20_Fact%20Sheets__Final_Shared_24_08_2022_0.pdf.

[7] Malleshappa K, Krishna S, Shashikumar Shashikumar (2012). Awareness and attitude of youth toward HIV/AIDS in rural Southern India. Biomed Res.

[8] (2021). National AIDS Control Organization. District-level HIV estimates and prioritization in India. India.

[9] Kariya P, Shringarpure KS, Patel AG (2023). Awareness and knowledge of HIV/AIDS in school going children of Surat, Gujarat. Int J Community Med Public Health.

[10] (2006). National AIDS Control Organization. Ministry of Health and Family Welfare: state wise HIV prevalence (1998-2004). https://www.aidsdatahub.org/sites/default/files/resource/india-facts-figures-hiv-estimates-2003.pdf.

[11] (2023). Report on the global HIV /AIDS epidemic. https://thepath.unaids.org/wp-content/themes/unaids2023/assets/files/2023_report.pdf.

[12] Carey MP, Schroder KE (2002). Development and psychometric evaluation of the brief HIV Knowledge Questionnaire. AIDS Educ Prev.

[13] Yadav SB, Makwana NR, Vadera BN, Dhaduk KM, Gandha KM (2011). Awareness of HIV/AIDS among rural youth in India: a community based cross-sectional study. J Infect Dev Ctries.

[14] Singh A, Jain S (2009). Awareness of HIV/AIDS among school adolescents in Banaskantha district of Gujarat. Health Popul Perspect Issues.

[15] Kawale SK, Sharma V, Thaware PP, Mohankar AD (2017). A study to assess awareness about HIV/AIDS among rural population of central India. Int J Community Med Public Health.

